# Which Are the Most Suitable Stents for Interventional Endoscopic Ultrasound?

**DOI:** 10.3390/jcm9113595

**Published:** 2020-11-08

**Authors:** Se Woo Park, Sang Soo Lee

**Affiliations:** 1 Division of Gastroenterology, Department of Internal Medicine, Hallym University Dongtan Sacred Heart Hospital, Hallym University College of Medicine, Hwaseong-si 18450, Korea; mdsewoopark@gmail.com; 2 Department of Gastroenterology, Asan Medical Center, University of Ulsan College of Medicine, Seoul 05505, Korea

**Keywords:** endoscopic ultrasound, drainage, therapeutic, intervention, stents

## Abstract

Endoscopic ultrasound (EUS)-guided interventions provide easy access to structures adjacent to the gastrointestinal tract, effectively targeting them for therapeutic purposes. They play an important role in the management of pancreatic fluid collections (PFC) and bile duct (BD) and pancreatic duct (PD) drainage in cases of failed endoscopic retrograde cholangiopancreatography (ERCP) or gallbladder (GB) drainage. Specially designed stents and delivery systems for EUS-guided transluminal interventions allow various new applications and improve the efficacy and safety of these procedures. In fact, EUS-guided drainage has emerged as the treatment of choice for the management of PFC, and recent innovations such as fully covered metal stents (including lumen-apposing metal stents) have improved outcomes in patients with walled-off necrosis. Similarly, EUS-guided BD and PD drainage with specially designed stents can be beneficial for patients with failed ERCP due to an inaccessible papilla, gastric outlet obstruction, or surgically altered anatomy. EUS-guided GB drainage is also performed using dedicated stents in patients with acute cholecystitis who are not fit for surgery. Although the field of dedicated stents for interventional EUS is rapidly advancing with increasing innovations, the debate on the most appropriate stent for EUS-guided drainage has resurfaced. Furthermore, some important questions remain unaddressed, such as which stent improves clinical outcomes and safety in EUS-guided drainage. Herein, the current status and problems of the available stents are reviewed, including the applicable indications, long-term clinical outcomes, comparison between each stent, and their future prospects.

## 1. Introduction

**Figure 1:**
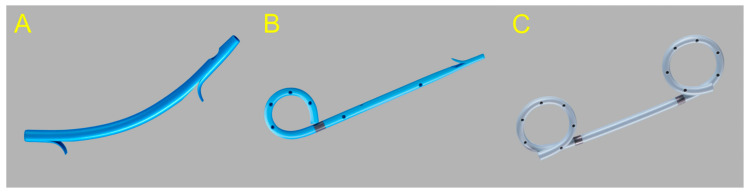
Plastic stents are made of polyethylene or Teflon and are available in varying sizes, shapes, and lengths for endoscopic ultrasound-guided drainage. (**A**) Straight “Amsterdam” type stent. (**B**) Single pigtail configuration helps anchor the stent for preventing inward migration. (**C**) Double pigtail configuration helps anchor the stent for preventing bidirectional migration.

**Figure 2:**
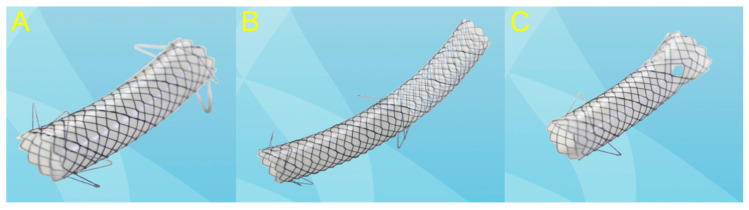
Various metal stents are available in varying sizes, shapes, and lengths for endoscopic ultrasound (EUS)-guided drainage. (**A**) A fully covered self-expandable metal stent with bidirectional anti-migrating flanges for transmural cyst drainage. (**B**) A partially covered self-expandable metal stent with bidirectional anti-migrating flanges for EUS-guided bile duct drainage. (**C**) A fully covered self-expandable metal stent with unidirectional anti-migrating flanges for transmural cyst drainage.

**Figure 3:**
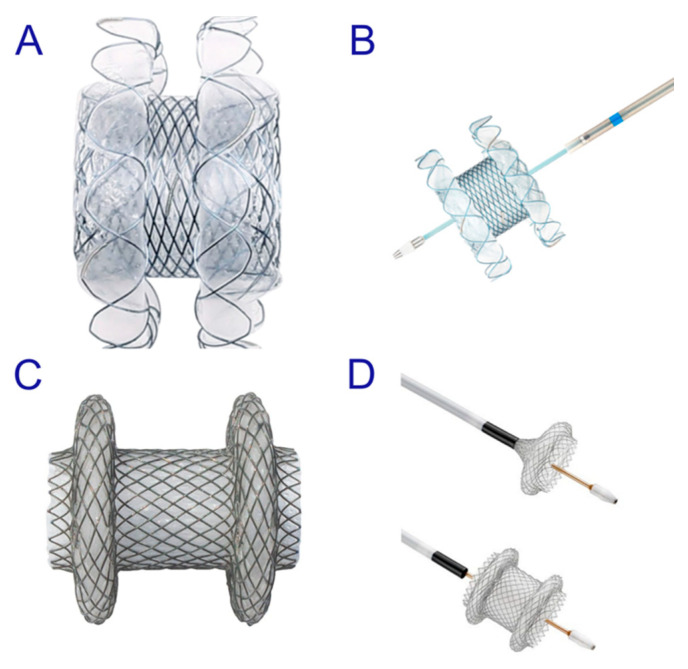
Lumen-apposing metal stents (LAMSs) have large diameters that facilitate both, drainage as well as access to extraluminal structures. These stents can appose two non-adherent structures, thereby minimizing the risk of migration and leakage. The Hot SPAXUS™ fully deployed (**A**) and with an electrocautery-enhanced delivery system (**B**). The Hot AXIOS™ fully deployed (**C**) and with an electrocautery-enhanced delivery system (**D**).

Endoscopic ultrasound (EUS)-guided drainage procedures are potentially disruptive alternatives to invasive surgery; thus, therapeutic strategies are undergoing a paradigm shift towards minimally invasive treatments, and a number of devices and techniques are being developed for easier and safer procedures. EUS-guided drainage procedures play an important role in the management of peripancreatic fluid collections (PFC) [1] and bile duct (BD) and pancreatic duct (PD) drainage in cases of failed endoscopic retrograde cholangiopancreatography (ERCP), gallbladder (GB) drainage, and entero-enteric anastomosis. In fact, recent innovations, such as the lumen-apposing metal stents (LAMS) designed specifically for EUS-guided interventions with bidirectional anchoring flanges, have improved outcomes in patients who require drainage [2,3,4]. Similarly, EUS-guided ductal drainage with a specially designed fully covered self-expandable metal stent (FCSEMS) or a partially covered SEMS (PCSEMS) can be beneficial for patients with failed ERCP, gastric outlet obstruction (GOO), or surgically altered anatomy [5,6]. Furthermore, EUS-guided GB drainage using LAMSs or dedicated SEMSs with bidirectional anti-migratory flanges is not only safe and reliable for acute cholecystitis, but also improves the quality of life in patients who are barely fit for surgical treatment [7,8,9]. Research on dedicated stents for interventional EUS is rapidly advancing with an increasing number of innovative and refined techniques. This review focuses on the advantages and disadvantages of the currently available stents for EUS-guided drainage. It also discusses the selection of suitable stents, including the plastic stent (Figure 1), SEMS (Figure 2), and LAMS (Figure 3), for each EUS-guided intervention.

## 2. Selection of Appropriate Stents for Endoscopic Ultrasound (EUS)-Guided Drainage Procedures

### 2.1. Stent for EUS-Guided Peripancreatic Fluid Collection (PFC) Drainage

**Figure 4:**
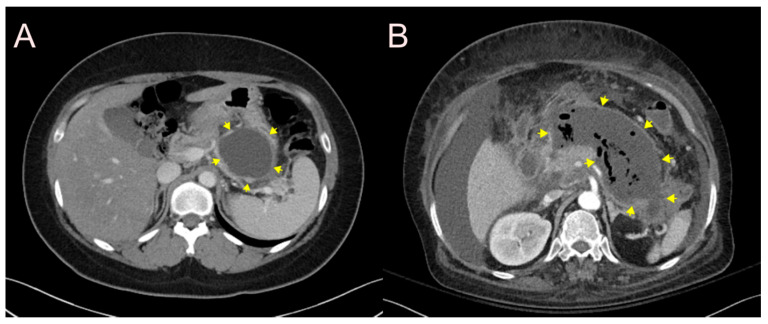
(**A**) A pseudocyst is defined as an encapsulated collection of fluid with a well-defined inflammatory wall, and is usually located outside the pancreas with minimal or no necrosis (yellow arrows pointing at the borders of the pseudocyst). (**B**) A walled-off necrosis (WON) is defined as a mature, encapsulated collection of peripancreatic necrosis that has developed a well-defined inflammatory wall with heterogeneous, non-liquid components in the retroperitoneum (yellow arrows pointing at the borders of the WON).

According to the revised Atlanta classification (proposed by a recent international consensus) [10], a pseudocyst is defined as a fluid collection that is surrounded by a well-defined wall. In essence, it contains fluid material, and not solid necrotic material (Figure 4A). On the other hand, walled-off necrosis (WON) is defined as a collection of necrotic pancreatic parenchyma and/or necrotic peripancreatic tissues encapsulated by a well-defined inflammatory wall; its maturation is usually completed after ≥4 weeks from the onset of necrotizing pancreatitis (Figure 4B). Based on this, an appropriate stent should be selected according to the characteristics and size of the PFC, proportion of solid debris, and thickness of the matured wall.

#### 2.1.1. Use of the Plastic Stent

**Figure 5:**
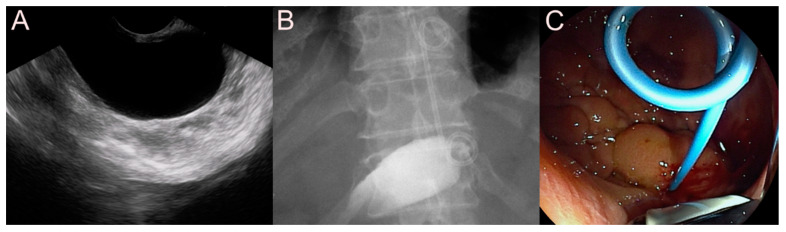
Endoscopic ultrasound (EUS)-guided pancreatic fluid collection (PFC) drainage using the 7 Fr double-pigtail plastic stent (DPPS) for a pseudocyst. (**A**) Endosonographic image showing a pseudocyst without sludge and heterogeneous debris. (**B**) Fluoroscopic image showing the DPPS placed between the stomach and the pseudocyst. (**C**) Endoscopic image showing the drainage of dark brown fluid materials and minimal sludge through the EUS-guided PFC drainage.

Generally, a 7 French (Fr) or a dedicated 8.5 Fr plastic stent can be used while a 10 Fr plastic stent is not suitable, because the working channel diameter in a linear echoendoscope is 3.7 or 3.8 mm (Figure 5). Regarding the type of plastic stent, a double-pigtail configuration is the only acceptable option for preventing bidirectional migration. In addition, side holes are not needed because the fluid in the cyst can leak into the peritoneum through the side hole. The stent length is determined by approximating the distance between the furthermost end of the PFC and the bowel wall, with an extra length of approximately 20 mm considered at both sides [11]. In case of a transgastric approach, the stomach wall may not tightly align with the cystic wall due to the patient’s position (supine or erect) and the amount of intragastric air, fluid, or food material [12]. This can lead to the stent’s inner migration into the cyst, outer migration to the stomach, or dislocation to the peritoneal cavity, resulting in cystic fluid leakage. Consequently, the selection of a double-pigtail stent can help prevent unintentional stent migration, particularly to the stomach. In terms of stent diameter, the plastic stent has a critical limitation: It has a relatively small luminal diameter, which can frequently predispose to stent occlusion, ineffective drainage, secondary infection, and the need for subsequent reinterventions [13,14]. Thus, the placement of multiple stents should facilitate adequate drainage. [15] However, such placement into the PFC is technically challenging and time-consuming, particularly when using a double-pigtail plastic stent (DPPS) with a diameter of more than 10 Fr [16].

In the case of PFC drainage, plastic stents are generally considered to be associated with a higher risk of recurrence or secondary infection as compared to the FCSEMS or LAMS, because the caliber of the plastic stent may be too small for sufficient drainage; thus, frequent revision for stent dysfunction may be required in EUS-guided PFC drainage with a plastic stent as opposed to a FCSEMS or LAMS. However, evidence from relevant studies indicated that the recurrence rate was similar between the stents, and instead of the stent type, PFC derived from disconnected PD syndrome or chronic pancreatitis were recognized as strong risk factors for recurrence [17,18,19].

#### 2.1.2. Use of the Self-Expandable Metal Stent (SEMS)

**Figure 6:**
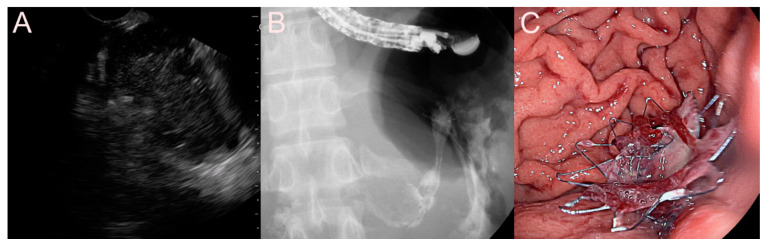
Endoscopic ultrasound-guided pancreatic fluid collection drainage using the dedicated bi-flanged, fully covered self-expandable metal stent (FCSEMS) (BONA-AL Stent; Standard Sci Tech Inc., Seoul, Korea) for a walled-off necrosis (WON). (**A**) Endosonographic image showing a WON with heterogeneous necrotic debris. (**B**) Fluoroscopic image showing the FCSEMS placed between the stomach and the WON. (**C**) Endoscopic image showing the drainage of pus-like materials and large amounts of sludge through the FCSEMS.

To overcome the theoretical limitations of the plastic stent, a dedicated bi-flanged FCSEMS was introduced due to its advantage of allowing a larger-diameter drainage, which can improve the patency of the stent, reduce secondary infections and, consequently, reduce the frequency of revisions for stent dysfunction and sustain the tract for sequential sessions of direct endoscopic necrosectomy (DEN) (Figure 6) [15]. A recent study demonstrated that the clinical success rate was 94% for a dedicated FCSEMS and only 74% for a plastic stent [13], although both stents achieved technical success in all cases. Another study on 230 patients who underwent FCSEMS or plastic stent placement found that the FCSEMS was associated with significantly higher clinical success rates and lower adverse event (AE) rates than the plastic stent. Moreover, in a recent network meta-analysis [20] of 15 studies comprising 1746 patients, Park et al. reported that the FCSEMS was superior to the plastic stent in terms of clinical success and had a lower bleeding risk than the LAMS. They found that stent migration did not differ between any two groups compared. Although deployment is much more convenient and faster for the FCSEMS than for the plastic stent, the FCSEMS should not be used for EUS-guided PFC drainage routinely, given the fact that the costs of devices for the FCSEMS are significantly higher than those for the plastic stent, despite similar clinical success rates [21]. Furthermore, tubular-structured FCSEMSs without antimigratory flanges are in theory not recommended in most EUS-guided drainage procedures, including EUS-guided PFC drainage, due to concerns regarding bidirectional stent migration.

#### 2.1.3. Use of the Lumen-Apposing Metal Stent (LAMS)

**Figure 7:**
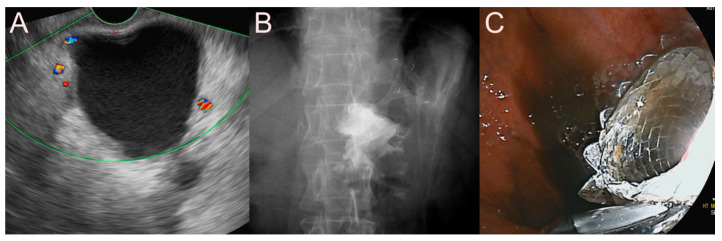
Ultrasound-guided pancreatic fluid collection drainage using the dedicated bi-flanged lumen-apposing metal stent (LAMS) (Niti-S SPAXUS; Taewoong Medical Co., Ltd., Ilsan, South Korea) for a walled-off necrosis (WON). (**A**) Endosonographic image showing a WON with heterogeneous necrotic debris. (**B**) Fluoroscopic image showing the LAMS placed between the stomach and the WON. (**C**) Endoscopic image showing the drainage of pus-like materials and large amounts of sludge through the LAMS.

The recently developed LAMS with bidirectional anti-migratory flanges (AXIOS [Boston Scientific, Marlborough, Massachusetts, USA] or Niti-S SPAXUS [Taewoong Medical Co., Ltd., Ilsan, South Korea]), which is specially designed for EUS-guided PFC drainage, has been found to be effective and safe (Figure 7) [2,3,4]. It can reduce the risk of migration and leakage by holding adjacent lumens in apposition. While AXIOS stents having diameters of 10 and 15 mm have been available in the market, the 20 mm AXIOS stent is newly released. The AXIOS and SPAXUS stents recently underwent efficiency improvements, and subsequently, the HOT AXIOS and HOT SPAXUS stents were released on the market. In these, an electrocautery tip was added to the distal end of the delivery system for transmission of the cutting current, thereby enhancing the easy passage of the stent through the walls without the need for prior tract dilation. Moreover, the LAMS simplifies DEN for WON by allowing free movement of the standard upper endoscope through its lumen, which may help shorten the procedure time and reduce the AEs associated with tract dilation whenever DEN is initiated [22,23]. In particular, for WON with large amounts of necrotic debris the LAMS is preferable for a more effective and rapid drainage and when there is a possibility of DEN. Based on the theoretical advantages of the LAMS, physicians should consider the selection of LAMSs in cases of WON containing at least 10% solid debris with adequate liquefactive necrosis and fluid within the PFC. However, it should be noted that LAMS is not suitable for a WON that mainly contains solid debris [24]. Furthermore, physicians should pay close attention to the size of the PFC and consider its relationships with the size of the inner anchoring flanges and with the length of the LAMS [21]. For example, for a 20 mm long SPAXUS stent having a diameter of 16 mm and equipped with a flange of diameter 25 mm, the safety depth of the PFC cavity should be at least 5 cm to facilitate easy deployment of the inner flange and prevent the impact of the LAMS on the opposite PFC wall [24]. Furthermore, one of the important factors affecting the technical and clinical success of the LAMS is the shape of the PFC. In a huge but shallow PFC having a vertical length of less than 2 cm, placement of multiple plastic stents, instead of the LAMS, may be preferred because plastic stents can be maneuvered to the desired direction easily for appropriate drainage, even in a cyst with acute angulation [15].

Regarding AEs, a recent network meta-analysis [20] reported that the risk of post-procedural bleeding was significantly higher for the LAMS than for the FCSEMS and the plastic stent. Bleeding during LAMS placement for PFC drainage is hypothesized to be attributed to the rapid shrinkage of the cyst immediately after LAMS insertion and to the unexpected inflow of large amounts of gastric acid [14]. Another hypothesis states that the plastic stent or FCSEMS may migrate spontaneously to the outer side as the cyst collapses, whereas the LAMS may remain in position and lead to mechanical injury against the regional vascular structures of the cystic wall [17]. Furthermore, the LAMS may have a lower risk of stent migration, because it has a theoretical advantage of having a saddle-shaped design due to wide anti-migratory flanges, which helps prevent bidirectional migration. While the FCSEMS also have anti-migratory flanges, these are not enough to anchor and prevent migration. Table 1 summarizes the possible subtypes, pros, and cons of each stent for EUS-guided PFC drainage.

### 2.2. Stent for EUS-Guided Bile Duct (BD) Drainage

In essence, EUS-BD drainage is categorized as EUS-guided hepatico-gastrostomy (HGS) (in which the intrahepatic duct [IHD] is drained mainly via a transgastric approach) and EUS-guided choledochoduodenostomy (CDS) (in which the extrahepatic duct [EHD] is drained mainly via a transduodenal approach). The selection of the approach is heavily dependent on the presence of GOO, level of ductal obstruction, and diameter of the dilated IHD. Previously, EUS-BD drainage was performed using a tubular FCSEMS, which was an efficient alternative to conventional trans-papillary stenting. However, the FCSEMS has a high risk of stent migration (with migration occurring in 27% of the cases), which can result in fatal AEs, including bile leakage with peritonitis. Several modifications based on the tubular configuration have been made to prevent migrations and dysfunction. Recently, a novel PCSEMS (hybrid metal stents; Standard Sci Tech Inc., Seoul, South Korea) featuring bidirectional anti-migratory flanges was introduced for EUS-BD drainage, and preliminary studies [5,25,26] have reported positive results with technical and clinical success rates of 100% and 86%, respectively, in patients who underwent EUS-guided HGS [27]. The plastic stent and a dedicated SEMS can be reasonable options for EUS-BD drainage. In theory, stent patency seems to be longer in EUS-guided BD drainage than in trans-papillary drainage, because the route in EUS‑guided BD drainage is free from the malignant stricture. Stent patency in EUS-BD drainage widely ranges from 62 to 402 days, [6] but it may be associated with a slight possibility of tumor-related ingrowth or overgrowth. Most cases of stent dysfunction that shorten the stent patency can occur from stent migration or clogging by food materials [28].

#### 2.2.1. Use of the Plastic Stent

**Figure 8:**
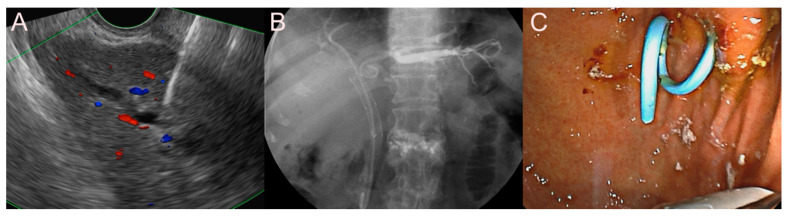
Ultrasound-guided hepatico-gastrostomy using the 7 Fr double-pigtail plastic stent (DPPS) for the obstruction of the left intrahepatic duct (IHD). (**A**) Endosonographic image showing a 19-gauge needle puncture into the left dilated IHD. (**B**) Fluoroscopic image showing the 7 Fr DPPS placed between the stomach and the left IHD. (**C**) Endoscopic image showing the drainage of pus-like materials through the 7 Fr DPPS.

Plastic stents with a double- or single-pigtail configuration are potential and effective options for preventing inner migration (Figure 8). Furthermore, side holes are required in the proximal and distal sides of the stent, but not in the stent body, because the bile juice is likely to leak through the side hole into the peritoneum. The stent length is determined by approximating the distance between the target IHD, hilum, or right side opposite to the IHD and the bowel, with an extra length of approximately 20 mm considered at the outer side. A 7 Fr DPPS can be a possible option due to greater accessibility and affordability, and is commercially available worldwide. In Japan, Itoi et al. developed a dedicated 8 Fr single-pigtail plastic stent (SPPS) with two flanges at each of the distal and proximal ends [29]. In theory, this modified, cost-effective stent would prevent stent migration secondary to stent shortening, a mechanical property in which the length of the SEMS shortens with stent expansion due to a radial force [29,30]. Umeda et al. [29] reported that the stent occlusion rate was 13.7% with a median stent patency of 4 months over a 5-month median follow-up period. Furthermore, another study indicated that endoscopic revision is relatively easy after a fistula has been created [31]. Unfortunately, this dedicated SPPS is not commercially available out of Japan. Both 7 and 8 Fr plastic stents are useful for a non-dilated IHD, whereas the SEMS can compress the IHD wall by the radial force itself. However, most plastic stents have a push-type deployment system; thus, withdrawing or changing the stent is impossible.

#### 2.2.2. Use of the SEMS

In theory, the use of the SEMS may be advantageous because it: (1) offers better drainage efficacy due to a diameter that is larger than that of the plastic stents, (2) prevents bile leakage or peritonitis, and (3) has a tamponade effect on tract bleeding due to compression derived from the radial force of the stent [32,33]. However, the SEMS may have limitations such as high costs, a shortening rate ≥ 40% for all braided-type stents, risk of fatal AEs (for, e.g., unexpected migration), and a direct obstruction of the side branches of the IHD [34,35]. Furthermore, stent mispositioning during deployment, particularly in a braided-type SEMS, is problematic during EUS-guided HGS drainage. Contrary to theoretical belief, several studies [25,36,37] have reported a higher incidence of bile peritonitis and pneumoperitoneum in patients who underwent SEMS placement, despite the presence of bidirectional flaps on the proximal and distal ends of the covered portion for prevention of stent migration.

**Figure 9:**
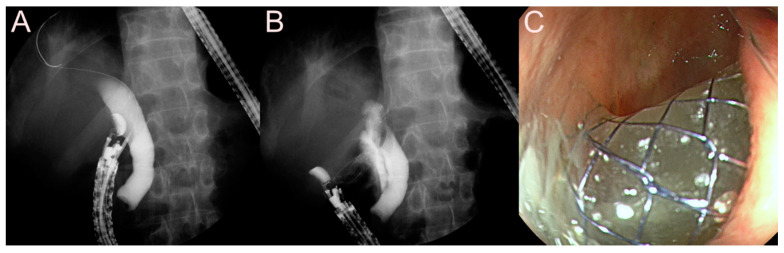
Ultrasound-guided choledochoduodenostomy using the bi-flanged, fully covered self-expandable metal stent (FCSEMS) (BONA-AL Stent; Standard Sci Tech Inc., Seoul, Korea) for distal malignant biliary obstruction. (**A**) Fluoroscopic image showing a guidewire delivered to the intrahepatic duct through a 19-gauge needle punctured into the common bile duct (BD). (**B**) Fluoroscopic image showing the bi-flanged FCSEMS placed between the duodenum and the common BD. (**C**) Endoscopic image showing the drainage of pus-like materials through the bi-flanged FCSEMS.

For EUS-guided CDS, a 6, 8, or 10 mm tubular stent can be used based on the diameter of the dilated EHD (Figure 9). The stent length is determined by approximating the distance between the puncture site (usually the duodenal wall) and the hilum, although a stent length of 6 cm is generally appropriate because the duodenal wall (fistula site) is positioned at the center of the stent with an adequate length in the duodenal lumen. [38] If the stent length is too short (i.e., <4 cm), it can be difficult to align the duodenal wall (fistula site) with the central portion of the stent. Conversely, if the stent is too long (i.e., ≥8 cm), the side branch of the IHD can be occluded. For EUS-guided CDS, the PCSEMS is not recommended due to the possibility of bile leakage, which may occur if the uncovered portion of the stent is positioned directly in the free space between the duodenum and the BD [39]. Considerable factors, including the structure of the stent, are important for stent selection. Stent structures are classified into the braided and laser-cut types; the braided-type stent has a high shortening rate (>40%), which can lead to inappropriate stent deployment or dislocation and even migration.

**Figure 10:**
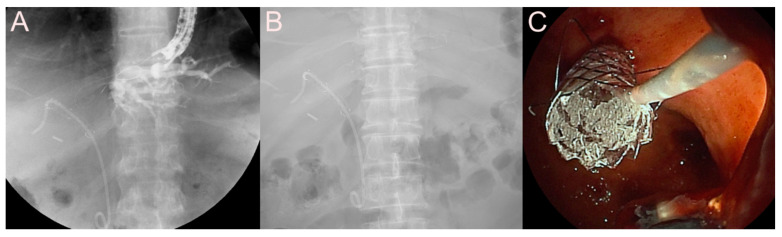
Ultrasound-guided hepatico-gastrostomy using the dedicated partially covered self-expandable metal stent (PCSEMS) (hybrid metal stents; Standard Sci Tech Inc., Seoul, Korea) for the obstruction of the left intrahepatic duct (IHD). (**A**) Fluoroscopic image showing a 19-gauge needle puncture into the left dilated IHD. (**B**) Fluoroscopic image showing the dedicated PCSEMS placed between the stomach and the left IHD. (**C**) Endoscopic image showing the drainage of pus-like materials through the dedicated PCSEMS.

For EUS-guided HGS, a 6 or 8 mm tubular stent, based on the diameter of the dilated IHD, may be preferable (Figure 10). Contrary to EUS-guided CDS, the FCSEMS cannot be recommended in EUS-guided CDS due to the possibility of stent-related occlusion of the side branch adjacent to the target IHD. Determination of the stent length is the most important factor in EUS-guided HGS. To determine the proper stent length, physicians should consider the distance from the target IHD to the gastric wall with an extra length of more than 20 mm at the gastric wall; this extra length should be determined on the basis of the stent structure (braided or laser-cut type). When using a laser-cut-type, the extra stent length should be at least more than 10 mm, while for the braided-type, the extra length should be longer than 20 mm considering the higher shortening rate of the stent itself. Several modifications have been made to minimize stent migrations and dysfunction. Recently, a dedicated metal stent, specially designed and modified for EUS-guided HGS and containing bidirectional anchoring flanges (hybrid metal stents; Standard Sci Tech Inc., Seoul, Korea), was developed; preliminary data on this [5,25,26] appear to be promising. A recent study [27] demonstrated that a hybrid metal stent had technical and clinical success rates of 100% and 85.7%, respectively, in patients who underwent EUS-guided HGS. This suggests that modified stents, such as hybrid metal stents, are effective in relieving biliary obstruction after failed ERCP and in reducing stent-related AEs, especially stent migration.

However, endoscopists have recognized the limitations of the SEMS, which include the possibility of stent migration that results from a high shortening rate of more than 40%, stent-related occlusion of the side branch adjacent to the target IHD, over-dilation of the narrow IHD, technical difficulty in stent deployment, and inappropriate positioning (in the case of a braided-type SEMS).

#### 2.2.3. Use of the LAMS

Several studies [40,41,42] have reported that EUS-guided CDS with LAMSs is efficacious and safe for distal malignant biliary obstruction. Kunda et al. [40] reported that the technical and clinical success rates in 127 EUS-guided CDS procedures involving the LAMS were 98.2% and 96.4%, respectively, without any occurrence of bile leakage. They concluded that EUS-guided CDS with LAMS could be the preferred method to perform a perpendicular puncture of the duct, especially when the EHD is larger than 15 mm in diameter and the stricture is located in the distal CBD. However, LAMS is not theoretically recommended in EUS-guided HGS due to concerns regarding the limited diameter of the IHD, which is not suitable for huge anchoring flanges. Table 2 summarizes the possible subtypes, pros, and cons of each stent for EUS-guided BD drainage.

### 2.3. Stent for EUS-Guided Gallbladder (GB) Drainage

Similar to EUS-guided BD drainage, EUS-guided GB drainage can be performed using either a transgastric or a transduodenal approach. It is important to identify an approach that achieves close anatomical apposition between the GB neck (as the target point) and the gastrointestinal tract (stomach or duodenum) and allows the maintenance of a stable echoendoscope position for the safe placement of the stent [43,44]. To date, there is a lack of evidence that supports the advantages and disadvantages of either approach. In general, the duodenum is a suitable puncture site because it is located in the retroperitoneum and has a close proximity to the GB neck for stable tract formation, while the stomach is located quite far from the GB neck and frequent peristaltic movements can lead to outward or inward stent migration into the GB with subsequent bleeding and recurrent cholecystitis [45,46,47]. Only in cases of potential candidates for subsequent cholecystectomy, the transgastric approach can be the preferable option due to a greater ease of gastric wall defect closure during cholecystectomy as compared to that of duodenal closure, despite a higher rate of AEs [48].

#### 2.3.1. Use of the Plastic Stent

**Figure 11:**
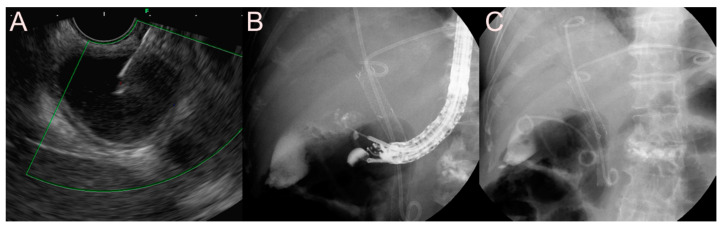
Endoscopic ultrasound-guided gallbladder (GB) drainage using the 7 Fr double-pigtail plastic stent (DPPS) for obstructive cholecystitis after biliary stent placement. (**A**) Endosonographic image showing a markedly dilated GB with large amounts of sludge and heterogeneous debris. (**B**) Fluoroscopic image showing a 19-gauge needle puncture into the GB neck portion. (**C**) Fluoroscopic image showing the 7 Fr DPPS placed between the duodenum and the GB.

Previously, similar to EUS-guided PFC or BD drainage, EUS-guided GB drainage was also performed using plastic stents (Figure 11) [49]. However, a tamponade effect by the hepatic parenchyma, which is seen with EUS-guided HGS, cannot be expected with EUS-guided GB drainage, because the interspace between the GB and the stomach/duodenum is the peritoneal free space [7]. Therefore, bile leakage can occur along the fistula adjacent to the plastic stent [50]. However, Jang et al. reported that no bile leakage or peritonitis occurred in patients who underwent EUS-guided GB drainage using a 5-Fr naso-biliary drainage tube. They assumed that bile leakage through the puncture site can be prevented by the adherence of an inflamed GB wall to the adjacent structures. In addition, the patency of the plastic stent is relatively shorter than that of the SEMS. Despite several limitations, a plastic stent can be a reasonable option if the patient is likely to consider sequential cholecystectomy in the future. In the same study [7], they also demonstrated that laparoscopic cholecystectomy was successfully conducted in 23 of the 29 patients (79.3%) who underwent EUS-guided GB drainage, and only two patients required conversion to open cholecystectomy. The authors concluded that EUS-guided GB drainage does not result in significant adhesions or inflammation between the GB and the duodenum that may otherwise be impedimental for cholecystectomy. Therefore, laparoscopic cholecystectomy can be performed safely without any technical difficulty, even in patients undergoing EUS-guided GB drainage.

#### 2.3.2. Use of the SEMS

**Figure 12:**
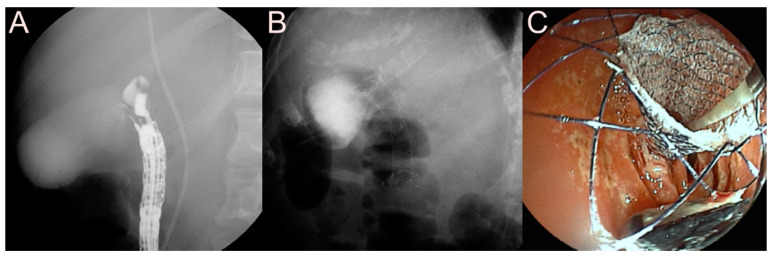
Endoscopic ultrasound-guided gallbladder (GB) drainage using the dedicated bi-flanged fully covered self-expandable metal stent (FCSEMS) (BONA-AL Stent; Standard Sci Tech Inc., Seoul, Korea) for cholecystitis. (**A**) The GB is punctured using a 19-gauge needle, and the contrast medium is injected. (**B**) Fluoroscopic image showing the dedicated bi-flanged FCSEMS placed between the duodenum and the GB. (**C**) Endoscopic image showing the drainage of pus-like materials through the dedicated bi-flanged FCSEMS.

As mentioned earlier, the SEMS has the theoretical advantage of having a larger diameter as compared to plastic stents and can be used in EUS-guided GB drainage, even in patients who are unfit for surgical cholecystectomy. First, it can prevent bile leakage between the stent and the fistula tract of the GB wall by self-expanding radial force, although small amounts of bile juice can leak during tract dilation before stent deployment. Therefore, the risk of bile peritonitis can be decreased clinically [51]. With the same mechanism, the SEMS can provide a tamponade effect, thereby enabling spontaneous hemostasis by the stent itself [7]. Second, the SEMS can be withdrawn and recaptured if the outer sheath of the delivery system is not pulled until the red marker as the maximal limit point permitting reconfiguration. Thus, the endoscopist can easily readjust the stent position when stent mispositioning is expected before full deployment. Third, it has a larger diameter, which can prevent frequent clogging by food materials or sludge in the GB, and thus, promote longer stent patency [7]. However, conventional tubular SEMSs without anti-migratory flanges are associated with a higher risk of stent migration and even bile leakage/peritonitis due to technical difficulties in achieving appropriate positioning during deployment. To overcome stent migration, the modified tubular SEMS with anti-migratory flanges was introduced by Lee et al. [9] This stent (BONA-AL Stent; Standard Sci Tech Inc., Seoul, Korea) was a PCSEMS containing a nitinol wire covered by a silicone membrane (Figure 12). These stents were 10 mm in diameter and 4–7 cm in length (when the flares were enlarged; 22 mm external diameter), creating a 90° angulation. In previous studies [52,53], no patient actually experienced bile leakage and peritonitis. As another technique for preventing stent migration, several studies [7,54,55] have recommended the insertion of a combination of the DPPS within the SEMS, because the DPPS remains in the GB even if the SEMS migrates, and the maintained fistula can allow revision [7,54,55]. In a recent systematic review [50] of clinical outcomes in EUS-guided GB drainage according to the stent type, the overall rate of AEs was lower for the SEMS than for the plastic stent; thus, it may be preferable for preventing procedure-related AEs in patients who are not likely to undergo a future cholecystectomy.

#### 2.3.3. Use of the LAMS

**Figure 13:**
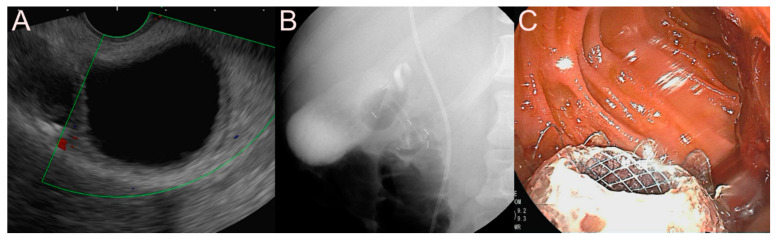
Endoscopic ultrasound-guided gallbladder (GB) drainage using the dedicated bi-flanged lumen-apposing metal stent (LAMS) (Niti-S SPAXUS; Taewoong Medical Co., Ltd., Ilsan, South Korea) for cholecystitis. (**A**) Endosonographic image showing a markedly dilated GB with large amounts of sludge and heterogeneous debris. (**B**) Fluoroscopic image showing the dedicated bi-flanged LAMS between the duodenum and the GB (distal flange of the LAMS deployed within the GB lumen). (**C**) Endoscopic image showing the drainage of pus-like materials through the dedicated bi-flanged LAMS in EUS-guided GB drainage (proximal flange of the LAMS in the duodenal bulb).

As mentioned earlier, the LAMS has been specially designed for procedures such as drainage or fistula formation, including PFC drainage [56], BD drainage (especially in EUS-guided CDS) [40], and creation of entero-enteric anastomosis [57]. In terms of EUS-guided GB drainage, the theoretical advantage of the LAMS over the plastic stent or tubular SEMS is the ability to apposition the GB wall tightly along the intestinal wall, which can prevent potential bile leakage by the sealing-off effect and inner or outer stent migration (Figure 13) [58]. Furthermore, it has a larger diameter, which can allow better efficacy of drainage. According to a recent report [59], the LAMS can be used for various transluminal interventions for intra-cholecystic pathologies, such as peroral cholecystoscopy using a magnifying endoscope or confocal endomicroscopy. In addition, interventional cholecystoscopy can be useful for GB stone removal with holmium laser lithotripsy.

During stent selection, physicians determine the diameter and length of the LAMS based on the anatomical position of the GB with respect to the duodenum/stomach, GB wall thickness or stiffness, or the size of the GB stones. [43] Several studies have demonstrated that the insertion of an additional plastic stent or a tubular SEMS in the LAMS could prevent stent occlusion or migration [47,58,60,61], particularly in patients in whom it is intended to remain in situ indefinitely. A recent systematic review reported that the pooled technical and clinical success rates of the LAMS were 95.2% and 96.7%, respectively. In terms of AEs, the rates of recurrence of cholecystitis, bleeding, and stent migration were acceptable at 5.1%, 2.6%, and 1.1%, respectively. Table 3 summarizes the possible subtypes, pros, and cons of each stent for EUS-guided GB drainage.

### 2.4. Stent for EUS-Guided Pancreatic Duct (PD) Drainage

Similar to EUS-guided HGS, EUS-guided PD drainage can be performed in two ways: the rendezvous technique (retrograde approach; stent placement is completed using a trans-papillary approach by exchanged side-viewing duodenoscope after obtaining PD access) and transmural drainage (antegrade approach; PD access and stent placement are completed as a one-step procedure using a linear echoendoscope). The approach route is selected in accordance with the accessibility to major or even minor papilla, level of PD stricture, indications of intervention, and diameter of the dilated PD. Furthermore, the optimized puncture site should be selected as the point located at the shortest distance from the stomach to the PD and should permit maximal stability of the echoendoscope for ensuring safe tract dilation and stent deployment, but have no intervening vessels. [62] According to most recent studies, the optimized PD approach was obtained using a transgastric approach. For EUS-guided antegrade PD drainage, fistula tract dilation should be performed using mechanical dilators (5–7 Fr tapered catheters or standard cannulas), hydrostatic balloons, and/or diathermic catheters such as a cystotome or needle-type knife. [63] To date, there have been no studies that compare the efficacy and safety of the various tract dilation techniques. The use of diathermic catheters still requires major consideration due to concerns regarding cautery-related AEs [64,65].

According to two major reasons for selecting the appropriate approach route, most endoscopists basically recommend that rendezvous technique be attempted first [66,67,68]. First, the rendezvous technique can facilitate natural and physiological drainage through the papilla or surgical anastomosis site for the decompression of PD obstruction. Second, compared to the rendezvous technique, transmural drainage (antegrade approach) frequently requires a sufficiently greater fistula tract dilation in each segment, including the PD wall, pancreatic parenchyma, and gastric wall, for the smooth delivery of the stent, which results in fatal AEs such as pancreatic juice leakage, pancreatitis, bleeding, or even perforation [69].

#### 2.4.1. Use of the Plastic Stent

**Figure 14:**
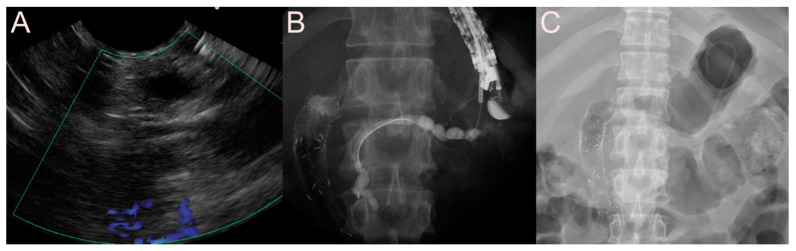
Endoscopic ultrasound-guided pancreatic duct (PD) drainage using the 7 Fr double-pigtail plastic stent (DPPS) for obstructive pancreatitis. (**A**) Endosonographic image showing a 19-gauge needle puncture into the dilated main PD. (**B**) Fluoroscopic image showing a guidewire delivered to the head portion of the main PD through a 19-gauge needle punctured into the upper site of the stomach. (**C**) Fluoroscopic image showing the 7 Fr DPPS placed between the main PD and the stomach.

Plastic stents with a straight, single, or double pigtail configuration can be placed at all stages of EUS-guided PD drainage (Figure 14). However, unlike in EUS-guided HGS, stents can easily migrate into the peritoneal cavity because the gastric wall and the pancreatic parenchyma are too close to each other [70]. Furthermore, the PD is usually not dilated sufficiently, and plastic stents can be adequate in this case [71]. A stent that tightly fits the diameter of the dilated tract may be suitable for preventing pancreatic juice leakage along the fistula tract and stent migration. Consequently, a 5 or 7 Fr plastic stent can be used in EUS-guided PD drainage. The need for side holes in the plastic stent is highly debatable due to concerns regarding the risk of pancreatic juice leakage, although a stent with multiple side holes is selected sometimes to avoid obstruction of the side branches. A biliary plastic stent without side holes is also another option for avoiding unexpected pancreatic juice leakage. Matsunami et al. [70] introduced a novel 7 Fr SPPS with a total length of 20 cm and effective allowable drainage length of 15 cm, which is applicable for recurrent pancreatitis derived from the PD or the pancreatico-enteric stricture. They demonstrated that the overall technical success rate was 100% without any reported stent migrations [72]. In one of the largest studies on EUS-guided PD drainage, although no significant difference was noted, stent migration tended to occur more frequently in straight-type plastic stent placement than in DPPS placement [65].

#### 2.4.2. Use of the SEMS

**Figure 15:**
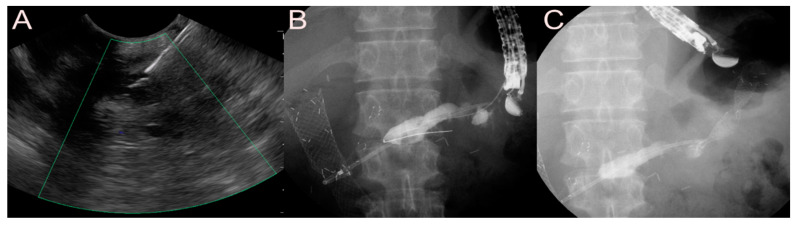
Endoscopic ultrasound-guided pancreatic duct (PD) drainage using the bi-flanged fully covered self-expandable metal stent (FCSEMS) (BONA-AL Stent; Standard Sci Tech Inc., Seoul, Korea) for obstructive pancreatitis. (**A**) Endosonographic image showing a 19-gauge needle puncture into the dilated main PD. (**B**) Fluoroscopic image showing a guidewire delivered to the head portion of the main PD through a 19-gauge needle punctured into the upper site of the stomach. (**C**) Fluoroscopic image showing the bi-flanged FCSEMS placed between the main PD and the stomach.

The FCSEMS is in theory not recommended due to concerns regarding the obstruction of the side branches of the PD, although it has several advantages for EUS-guided PD drainage. The covering membrane of the FCSEMS can lead to branch duct obstruction via cross-stream blockage of the main PD. Moreover, an uncovered SEMS is not recommended due to the risk of leakage between the stomach and pancreas and technical difficulties in withdrawal or replacement due to tissue hyperplasia and ingrowth [73]. Compared to the plastic stent, the SEMS has a larger diameter that facilitates adequate drainage, longer stent patency, and easy revision for stent dysfunction. Moreover, it can prevent bleeding or pancreatic juice leakage during tract dilation by the tamponade effect exerted by the radial force of the SEMS itself (Figure 15) [74]. Furthermore, compared to the plastic stent, it may maintain its position for a longer duration. To date, there have been few reports on the use of the SEMS for EUS-guided PD drainage because plastic stents have been used more commonly [75]. However, compared to a plastic stent, the SEMS may be more suitable for painful malignant main PD obstruction, because it may minimize the need for endoscopic revision for stent dysfunction, potentially without exchange, in patients who are expected to survive for longer than 6 months. In a study [76] on the use of the FCSEMS for EUS-guided PD drainage, Oh et al. demonstrated that the use of the novel FCSEMS (with anti-migration properties) achieved technical and clinical success rates of 100% in all 25 consecutive patients with painful obstructive pancreatitis. They also reported that no stent-related AEs, including migration, obstruction by food materials or clogging, or ductal stricture, were observed during the follow-up period of 221 days. Therefore, according to this study, the use of the FCSEMS may be technically feasible and relatively safe despite the lack of strong evidence supporting the use of SEMS for EUS-guided PD drainage. Furthermore, the use of the LAMS may be limited for EUS-guided PD drainage because of a non-dilated PD and the unique design of wide anchoring flanges. Table 4 summarizes the possible subtypes, pros, and cons of each stent for EUS-guided PD drainage.

### 2.5. Stent for EUS-Guided Creation of Entero-Enteric Anastomosis

**Figure 16:**
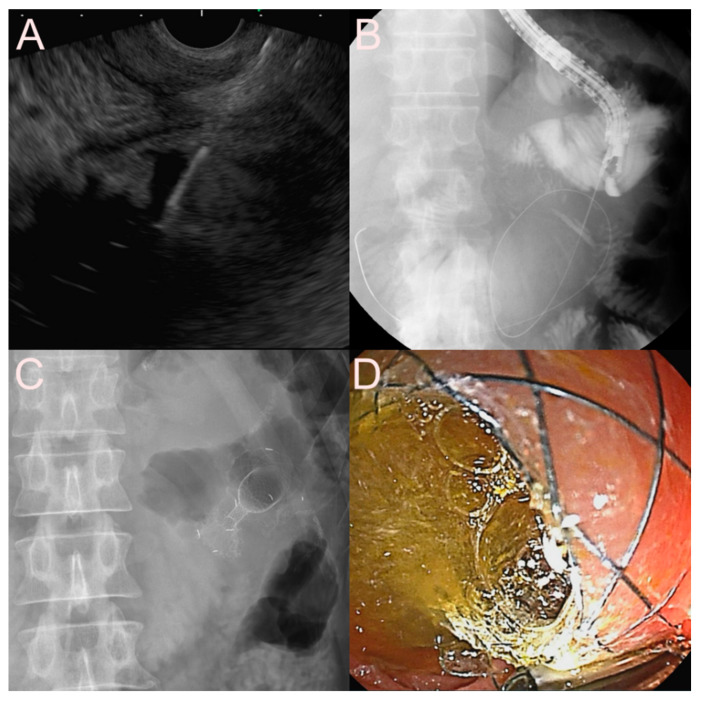
Ultrasound-guided jejunojejunostomy using the dedicated bi-flanged lumen-apposing metal stent (LAMS) (Niti-S SPAXUS; Taewoong Medical Co., Ltd., Ilsan, South Korea) for afferent loop syndrome in a patient who underwent total gastrectomy with Roux-en-Y anastomosis. (**A**) Endosonographic image showing a markedly dilated afferent loop with large amounts of sludge and heterogeneous debris. (**B**) Fluoroscopic image showing a guidewire delivered to the afferent jejunal loop through a 19-gauge needle punctured from the patent efferent jejunal loop. (**C**) Fluoroscopic image showing the dedicated bi-flanged LAMS placed between the afferent and efferent loops. (**D**) Endoscopic image showing the drainage of pus-like materials through the dedicated bi-flanged LAMS.

EUS-guided entero-enteric anastomosis creation has been applied to the management of the afferent loop syndrome or GOO [77,78,79]. The ideal stents for this procedure may include a bi-flanged LAMS (AXIOS or SPAXUS), which may provide long-term luminal patency for the free passage of food materials without surgical limitations (Figure 16). In the largest series [80] of 26 patients with GOO treated by EUS-guided gastrojejunostomy (GJ) creation using a LAMS, the technical success rate was 92%, whereas the clinical success rate (clinical success was defined as the tolerability of oral diet) was 85%. In another study, [81] Itoi et al. used a specially designed double-balloon enteric tube to create a window for transgastric puncture. In the EUS-guided creation of entero-enteric anastomosis, the most important factor, instead of the stent type, is the creation of a window for an initial transgastric puncture, which is categorized within the direct EUS-guided GJ (water-immersion technique) and the balloon-assisted EUS-guided GJ techniques.

For the water-immersion technique, a large volume of water should be infused into the proximal jejunum through the endoscope itself or a needle [79,80,81,82]. This facilitates the distention of the jejunal lumen and the appropriate creation of a window for transgastric puncture. Then, an electrocautery-enhanced LAMS (Hot AXIOS or Hot SPAXUS) is used to simultaneously puncture the dilated jejunal lumen and deploy the LAMS in a one-step procedure. In contrast, in the balloon-assisted technique, balloon dilation should be performed after needle puncture to allow the passage of the 10.8 Fr stent delivery system over the guidewire transgastrically into the jejunum in a two-step procedure [83].

The dilating nasojejunal balloon is necessary for the balloon-assisted technique. The balloon catheter should be passed into the jejunum over a guidewire and inflated with water as a window for transgastric puncture, instead of a bolus of water, in the balloon-assisted technique [81]. If the inflated balloon is positioned properly under EUS guidance, a transgastric puncture using the electrocautery-enhanced LAMS or a 19-gauge needle (for the non-cautery-enhanced version) is performed to burst the balloon [79]. The subsequent puncture and stent deployment are then performed using the one- or two-step procedure described previously.

In another application of the LAMS for entero-enteric anastomosis, recent trials have been focused on obtaining gastrostomy access to the excluded stomach in patients who have undergone a Roux en Y gastric bypass (which poses distinct challenges for performing ERCP) [84]. Several studies [84,85,86] reported that the internal EUS-directed transgastric ERCP (EDGE) procedure may offer an effective and minimally invasive option for a common problem in an altered anatomy. This method facilitates endoscopic creation and reversal of a gastro-gastric fistula or a jejuno-gastric fistula using a LAMS through which ERCP or other endoscopic intervention can be performed. However, there are some concerns regarding this method; these include the risk of stent migration and subsequent peritonitis and weight regain after creating the fistula [87].

**Figure 17:**
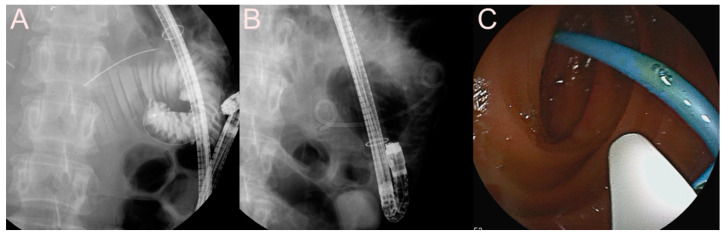
Double-balloon-assisted jejunojejunostomy using the 7 Fr double-pigtail plastic stent (DPPS) for the afferent loop syndrome in a patient who underwent total gastrectomy with Roux-en-Y anastomosis. (**A**) Fluoroscopic image showing a markedly dilated afferent loop with large amounts of sludge and heterogeneous debris. (**B**) Fluoroscopic image showing the 7 Fr DPPS placed between the afferent and efferent loops. (**C**) Endoscopic image showing the drainage of pus-like materials through the 7 Fr DPPS.

If the main purpose of the EUS-guided creation of entero-enteric anastomosis is only the drainage of the obstructive enteric loop not feeding in a situation, such as in the afferent loop syndrome, DPPS can be a possible option (Figure 17). Table 5 summarizes the possible subtypes, pros, and cons of each stent for EUS-guided creation of entero-enteric anastomosis.

## 3. Conclusions

EUS-guided interventions, including the drainage procedure as a minimally invasive treatment modality, have been well-utilized in an extended variety of clinical situations beyond their traditional application in simple PFC drainage. Despite the improvements made to various stents, selecting the optimal stent in a specific intervention for adequate drainage remains clinically challenging. Therefore, when performing EUS-guided drainage, it is important to consider the stent’s characteristics, anatomical properties of the target lesions, and potential stent-related AEs. Moreover, the stents available in the market have shown great adaptability; however, further research on new developments of dedicated stents is needed, which can increase the scope of their utilization and maximize their suitability for customized strategies of specific EUS-guided drainage.

## Figures and Tables

**Table 1:** Comparison of the characteristics of each stent for endoscopic ultrasound (EUS)-guided pancreatic fluid collection (PFC) drainage.

	Possible Subtype	Advantages	Disadvantages
**Plastic stent**	- Double-pigtail configuration	- Cheap - Easy to revise	- Frequent stent occlusion - Rare but possible leakage
**Fully covered self-expandable metal stent (FCSEMS)**	- FCSEMS with anti-migrating flanges	- Longer stent patency as compared to plastic stents - Theoretically lesser leakage	- Expensive - Stent malposition or migration
**(Lumen-apposing metal stent) LAMS**	- LAMS with bidirectional anchoring flanges - LAMS with or without electrocautery-enhanced tip	- Largest diameter - Longer stent patency - Suitable for direct endoscopic necrosectomy - Suitable for fistula creation	- Too expensive - Rare adverse events of bleeding or buried LAMS syndrome

**Table 2:** Comparison of the characteristics of each stent for endoscopic ultrasound (EUS)-guided bile duct (BD) drainage.

	Possible Subtype	Advantages	Disadvantages
**Plastic stent**	- Single-pigtail configuration - Double-pigtail configuration	- Cheap - Easy to revise - Free from stent shortening - Free from tumor ingrowth/overgrowth	- Frequent stent occlusion - Rare but possible leakage
**Fully covered self-expandable metal stent (FCSEMS)**	- FCSEMS without anti-migrating flanges - PCSEMS or FCSEMS with anti-migrating flanges (unidirectional or bidirectional)	- Longer stent patency than plastic stent - Theoretically lesser leakage	- Expensive - Side branch obstruction - Stent malposition or migration
**Lumen-apposing metal stent (LAMS)**	- LAMS with bidirectional anchoring flanges (for EUS-guided choledochoduodenostomy [CDS]) - LAMS with or without electrocautery enhanced tip (for only EUS-guided CDS)	- Largest diameter - Longer stent patency - Suitable for creation of fistula - Migration is rare	- Too expensive - Rare adverse events of bleeding or buried LAMS syndrome

**Table 3:** Comparison of the characteristics of each stent for endoscopic ultrasound (EUS)-guided gallbladder (GB) drainage.

	Possible Subtype	Advantages	Disadvantages
**Plastic stent**	- Double-pigtail configuration	- Cheap - Easy to revise - Free from stent shortening	- Frequent stent occlusion - Rare but possible leakage
**Fully covered self-expandable metal stent (FCSEMS)**	- FCSEMS with anti-migrating flanges (bidirectional)	- Longer stent patency than plastic stent - Theoretically lesser leakage	- Expensive - Stent malposition or migration
**Lumen-apposing metal stent (LAMS)**	- LAMS with bidirectional anchoring flanges - LAMS with or without an electrocautery-enhanced tip	- Largest diameter - Longer stent patency - Suitable for creation of fistula - Migration is rare	- Too expensive - Rare adverse events of bleeding or buried LAMS syndrome

**Table 4:** Comparison of the characteristics of each stent for endoscopic ultrasound (EUS)-guided pancreatic duct (PD) drainage.

	Possible Subtype	Advantages	Disadvantages
**Plastic stent**	- Straight configuration - Single-pigtail configuration - Double-pigtail configuration	- Cheap - Easy to revise - Free from stent shortening - Free from tumor ingrowth/overgrowth	- Frequent stent occlusion - Rare but possible leakage
**Fully covered self-expandable metal stent (FCSEMS)**	- FCSEMS without anti-migrating flanges - Partially covered self-expandable metal stent (PCSEMS) or FCSEMS with anti-migrating flanges (unidirectional or bidirectional)	- Longer stent patency than plastic stent - Theoretically lesser leakage	- Expensive - Side branch obstruction - Stent malposition or migration
**Lumen-apposing metal stent (LAMS)**	- LAMS is not recommended		

**Table 5:** Comparison of the characteristics of each stent for endoscopic ultrasound (EUS)-guided creation of entero-enteric anastomosis.

	Possible Subtype	Advantages	Disadvantages
**Plastic stent**	- Double-pigtail configuration (only for drainage purpose)	- Cheap - Easy to revise	- Frequent stent occlusion - Rare but possible leakage
**Fully covered self-expandable metal stent (FCSEMS)**	- FCSEMS with anti-migrating flanges (bidirectional) (only for drainage purpose)	- Longer stent patency than plastic stent - Theoretically lesser leakage	- Expensive - Stent malposition or migration
**Lumen-apposing metal stent (LAMS)**	- LAMS with bidirectional anchoring flanges - LAMS with or without an electrocautery-enhanced tip	- Largest diameter - Longer stent patency - Suitable for creation of fistula - Migration is rare	- Too expensive - Rare adverse events of bleeding or buried LAMS syndrome
